# Uncovering Molecular Features that Contribute to Tau Spread and Aggregation

**DOI:** 10.1002/alz.092904

**Published:** 2025-01-03

**Authors:** Jennifer Rauch

**Affiliations:** ^1^ University of Massachusetts‐Amherst, Amherst, MA USA

## Abstract

**Background:**

Tauopathies are a class of neurodegenerative diseases marked by tau protein spread and aggregation. Recently, our group described the cellular receptor Low‐density lipoprotein Receptor‐related Protein 1 (LRP1) as a regulator of tau spread. Knockdown of LRP1 halts tau spread in human induced pluripotent stem cell‐derived neurons and the mouse brain, indicating potential therapeutic implications. However, a comprehensive understanding of the tau‐LRP1 molecular complex remains elusive.

**Method:**

Our research focuses on elucidating the tau‐LRP1 structural interface and investigating how tau aggregate structure influences LRP1 processing. We are using covalent‐labeling mass spectrometry to map the tau‐LRP1 interface in vitro and in cells. We are isolating brain‐derived tau oligomers from various tauopathies and measuring their dependence on LRP1 using cell culture models of tau propagation.

**Result:**

Our results have identified residues critical for the interaction between tau and LRP1. Further, we have determined that brain‐derived tau oligomers utilize LRP1 for their endocytosis and that this is potentially dependent on structural elements of tau oligomers.

**Conclusion:**

Our work provides insights into the structural aspects underlying tau‐LRP1 interactions and lays the groundwork for the design of potential disease therapeutics.

